# Exercise-Induced Cognitive Improvement Is Associated with Sodium Channel-Mediated Excitability in APP/PS1 Mice

**DOI:** 10.1155/2020/9132720

**Published:** 2020-03-18

**Authors:** Ya-Xin Tan, Guang-Cai Liu, Hong-Lan Chen, Min-Nan Lu, Bo Chen, Tao Hu, Li Zhang, Rui Mao, Shan Li, Rong Mei, Xu-Yang Wang, Yan-Bin Xiyang

**Affiliations:** ^1^Institute of Neuroscience, Basic Medical College, Kunming Medical University, Kunming, Yunnan 650500, China; ^2^Department of Laboratory Medicine, The Third People's Hospital of Yunnan Province, Kunming, Yunnan 650011, China; ^3^Experiment Center for Medical Science Research, China; ^4^Editorial Department of Journal of Kunming Medical University, China; ^5^School of Stomatology, Kunming Medicine University, Kunming, Yunnan 650500, China; ^6^Department of Neurology, The First People's Hospital of Yunnan Province, Kunming, Yunnan 650032, China; ^7^Department of Neurosurgery, Shanghai Jiao Tong University Affiliated 6th People's Hospital, Shanghai, China 200233

## Abstract

Elevated brain activation, or hyperexcitability, induces cognitive impairment and confers an increased risk of Alzheimer's disease (AD). Blocking the overexcitation of the neural network may be a promising new strategy to prevent, halt, and even reverse this condition. Physical exercise has been shown to be an effective cognitive enhancer that reduces the risk of AD in elderly individuals, but the underlying mechanisms are far from being fully understood. We explored whether long-term treadmill exercise attenuates amyloid precursor protein (APP)/presenilin-1 (PS1) mutation-induced aberrant network activity and thus improves cognition by altering the numbers and/or distribution of voltage-gated sodium channels (Nav) in transgenic mice. APP/PS1 mice aged 2, 3.5, 5, 6.5, 8, and 9 months underwent treadmill exercise with different durations or at different stages of AD. The alterations in memory, electroencephalogram (EEG) recordings, and expression levels and distributions of Nav functional members (Nav1.1*α*, Nav1.2, Nav1.6, and Nav*β*2) were evaluated. The results revealed that treadmill exercise with 12- and 24-week durations 1) induced significant improvement in novel object recognition (NOR) memory and Morris water maze (MWM) spatial memory; 2) partially reduced abnormal spike activity; and 3) redressed the disturbed cellular distribution of Nav1.1*α*, aberrant Nav*β*2 cleavage augmentation, and Nav1.6 upregulation. Additionally, APP/PS1 mice in the 24-week exercise group showed better performance in the NOR task and a large decrease in Nav1.6 expression, which was close to the wild-type level. This study suggests that exercise improves cognition and neural activity by altering the numbers and distribution of hippocampal Nav in APP/PS1 mice. Long-term treadmill exercise, for about 24 weeks, starting in the preclinical stage, is a promising therapeutic strategy for preventing AD and halting its progress.

## 1. Introduction

The number of people aged 60 and over will increase from 222 million to 480 million from 2015 to 2050 [1]. The prevalence of age-related disorders, such as dementia and other cognitive impairments, is also expected to increase incrementally in worldwide. Alzheimer's disease (AD), as the leading cause of dementia among the elderly, usually induces cognitive deficits, which imposes a burden on the healthcare system and society [2].

Evidence from clinical observations and animal studies reveals that increased activation, or hyperexcitability, in the brain regions of memory networks is responsible for the development of amnestic mild cognitive impairment (aMCI) and AD, a debilitating and progressive type of dementia [3–6]. Although seizures were previously thought to be secondary to AD progression, aberrant activity and/or seizures may directly contribute to cognitive deficits early in the disease [7]. However, there are currently no available disease-modifying therapies, and the failure of several recent pathology-based strategies has highlighted the urgent need for novel effective therapeutic targets [8].

As a nonpharmacological therapy, physical exercise that targets modifiable risk factors and neuroprotective mechanisms has been shown to slow age-related decline and reduce disease-related cognitive impairment in the elderly [9]. Aerobic physical exercise has also been demonstrated to improve the performance of cognitively demanding tasks and reduce the risk of AD and Parkinson's disease in the elderly [10]. Exercise increases neuronal growth in aged rats [11], reduces hippocampal network hyperexcitability, and protects against seizure susceptibility in a rat model of epilepsy and depression comorbidity [12], which promotes recovery of learning and memory. Although much research showing that exercise can serve as a cognitive enhancer has been published and is of great interest in neuroscience, the mechanisms of cognitive improvement associated with exercise-induced hyperexcitability amelioration in the elderly are far from being fully understood.

Voltage-gated sodium channels (Nav), which are necessary for generating and propagating action potentials, play crucial roles in cell excitability [13–15]. Generally, a functional Nav is composed of a main pore-forming *α*-subunit associated with one or two accessory *β*-subunits [16]. Currently, ten *α*-subunits and four *β*-subunits have been authenticated and reported [15]. Nav1.1, Nav1.2, and Nav1.6 channel subtypes (each involving a different *α*-subunit) have been detected in the adult brain and are responsible for voltage-dependent sodium current regulation across the plasma membrane. Nav1.1 is primarily localized to the neuronal soma [17, 18], while Nav1.2 and Nav1.6 show preferentially high expression in axons [19, 20]. The *β*-subunits, which are type I single-transmembrane proteins, directly interact with the *α*-subunits and regulate their function, such as the localization, trafficking, and inactivation of Nav1.1 subunits [16, 21–23]. The *β*2-subunit (Nav*β*2), one of the four *β*-subunits, has been shown to play a major role in the regulation of the total and cell-surface density of sodium channels in neurons [24–26]. In a voltage-dependent manner, Nav*β*2 causes the activation and inactivation of the Nav1.1 and Nav1.6 channels [27, 28]. Dysregulation and diffuse distribution of Nav*β*2 along demyelinated axons and in brain neurons prevent conduction and induce aberrant neuronal activity [29, 30]. In summary, the maintenance of normal neuronal activity is dependent on the effective and precise cooperation between the functional subunits of sodium channels.

This study is aimed at investigating whether exercise-induced cognitive improvement is associated with reduced cell hyperexcitability via Nav regulation.

## 2. Materials and Methods

### 2.1. Ethical Approval

All the experiments and animal care complied with the Guide for the Care and Use of Laboratory Animals published by the US National Institutes of Health (publication no. 85-23; revised 1996). This study was performed in accordance with the Care and Use Guidelines of Experimental Animals established by the Research Ethics Committee of Kunming University of China (permit no. kmu-eac-2017008; Kunming, China). Anesthesia was performed using isoflurane for the surgical procedures. The purchase, storage, and use of the isoflurane were approved by Kunming Medical University (license no. kmykdx-D-A00272; Kunming, China). All efforts were made to minimize animal suffering during the experiments.

### 2.2. Animal Grouping

In this study, APPswe/PS1*Δ*E9 mice (amyloid precursor protein [APP]/presenilin-1 [PS1] transgenic mice with a C57BL/6J genetic background) were purchased from the Institute of Laboratory Animal Science (Chinese Academy of Medical Sciences, Beijing, China). The transgenes were confirmed by PCR genotyping of mouse tail tissue [31, 32]. Only female mice were utilized because female APP/PS1 mice develop cognitive deficits faster than the male mice [33].

Previous study used 18F-labeled fluorodeoxyglucose (FDG) microPET detecting the brains of APP/PS1 transgenic (TG) mice to evaluate age- and brain region-specific changes of glucose metabolism, which demonstrated that APP/PS1 mice showed intact cognition at age 2 months (m), learning deficits at 3.5 m, learning and memory deficits at 5 m, and further learning and memory impairments at 8 m [33]. It seems that APP/PS1 mice of 2 m mimic the preclinical stage of AD (prodromal-AD), whereas APP/PS1 mice of 3.5 m, 5 m, and 8 m exhibit subclinical, early clinical, and midclinical signs of AD (symptomatic-AD) [33]. As medium-/long-term exercise induces promising improvements in learning and memory [9, 34], we studied the impact of medium-/long-term treadmill exercise (for 12 and 24 weeks, respectively) on mice at different stages of AD. We employed 2 m, 3.5 m 5 m, 6.5 m, 8 m, and 9.5 m APP/PS1 mice to explore the possible effects of 12- and 24-week exercises on the age-specific alterations of memory and neuronal excitability related to AD progression, and we explored the associations between these alterations and Nav numbers and distribution. To investigate the optimum intervention time window for exercise, the mice in the 12-week exercise group began exercise at the prodromal-AD stage (2 m; designated the early 12-week group) or clinical AD stage (6.5 m; designated the late 12-week group).

The mice were housed with *ad libitum* access to food and water and exposed to a 12 h light/dark cycle. The mice were housed in a group and allowed to acclimatize to their environment for 1 week prior to commencement of the experiments. The mice in each age group were then divided into three duration subgroups: early 12-week (starting at 2 m), late 12-week (starting at 6.5 m), and 24-week. See comment above (starting at 2 m) subgroups. The APP/PS1 mice in each of these duration subgroups were divided into sedentary (control) and exercise cohorts (Table 1). Age-matched littermate wild-type (WT) mice with a C57BL/6J background were used as controls (Table 2). The mice in the exercise groups learned how to run on a horizontal treadmill for 15 min/day for 1 week before the start of the experiments.

### 2.3. Treadmill Exercise

The protocol for the treadmill exercise was adapted from previous studies [34, 35] with modifications. The treadmill (Xinruan Technology Co., Ltd, Hangzhou, China) was equipped with wire loops and retention sensors, as described previously, to deliver a mild electric shock (at 0.6 mA, with an interpulse interval of <2 s) [35], which forced the mice to run at the required speed (15 m/min). Each mouse was forced to exercise for 60 min/day at a speed of 15 m/min at some point from 7 to 9 p.m. and then allowed to recover for an additional 12 h. Each mouse was allocated a rest period of 1.5 min every 30 min during the treadmill exercise [35]. The mice in the exercise groups were subjected to treadmill exercise 5 days per week for 12 or 24 weeks.

### 2.4. Open Field Assessment

To assess the gross locomotor and exploratory activity of the mice in each group, mice were placed in a brightly lit circular arena (40 × 40 × 40 cm^3^) to assess their general activity levels. The horizontal (entire distance traveled) and vertical (number of rearing movements) activity were recorded and quantified using the TOPSCAN tracking system (Clever Sys Inc., USA) over a 10-minute trial period. TopScan animal behavior analysis system is a set of software and hardware system which automatically tracks and records animal activity and behavior and calculates various behavioral indexes by video tracking and computer, using image processing technology.

### 2.5. Novel Object Recognition (NOR) Task

NOR tasks were performed in the same arena (40 × 40 × 40 cm^3^) used for the open field assessment, as previously described [36, 37]. Test objects (4 or 5 cm in height) were made of glass or plastic and had different shapes, textures, colors, and sizes. During the initial training period of 5 min, mice were placed at the center of the arena with two identical objects. The amount of time exploring each object was recorded independently by trained researchers. At 1 h post training, the mice were reinserted into the arena for the test session for 10 min. During this session, one of the objects was replaced by a novel one, which was different in shape and color but consistent in height. The exploration of the object was defined as direct contact of the nose or front paws with the object. Again, the amount of time spent exploring the familiar and novel objects was recorded. Animals that had a total exploration time < 8 s were excluded from the NOR tests. Results are expressed as the discrimination index (DI), which is defined as the explorative time for the new object divided by the total explorative time for both objects during the training and test session. Results about the total object exploration time are in Table 3.

### 2.6. Morris Water Maze (MWM) Test

The MWM consisted of a circular pool (100 cm in diameter, 50 cm deep) filled with white water at 24 ± 1°C to a depth of 20 cm. The MWM test was run as previously described [32]. All mice carried out a preliminary experiment, which was to adapt them to the water and allow them to step on a white platform (hidden below the water surface) and escape from the water, the day before the regular testing. For this experiment, a mouse placed in the water was allowed to swim around for 10 s and then placed on a white platform submerged under water for only 1 to 2 s. For regular testing, the hidden platform was put in a different location. The time mice found the hidden submerged white platform was recorded as escape latency and recorded for 5 days in a row. On testing day 6, the platform was removed for the probe trial, and each mouse was allowed to swim for 60 s to assess their memory for the platform location. The percentages of time spent in the target quadrant, path in the target quadrant, number of target platform crossings, and swim speed (mm/s) were recorded. The time spent and distances traveled in the four quadrants were noted. The animals were tracked with an overhead video camera, and the data was analyzed by an animal behavior video analysis system.

### 2.7. Electroencephalogram (EEG) Recordings

(a) Electrode implantation. For EEG recordings, a high resolution mouse EEG using polyimide-based microelectrode array (PBM-array) was used in the study. The protocols of electrode implantation and EEG recordings were performed according to the literature with some modifications [7, 38, 39]. Mouse was fixed on the stereotaxic instrument after anesthetized for electrode fixation before EEG recordings. First, middle scalp was incised about 2 cm to expose the skull and found the bregma and lambda points (recognized electrode position on the surface of the mouse brain) on the skull according to the mouse brain atlas (Paxinos and Franklin's the Mouse Brain in Stereotaxic Coordinates). Then mice were implanted a triple wire electrode with bare tips (1.0 mm in diameter), consisting of three polyimide-coated wires with bare tips, in the hippocampus (AP − 2.1 mm, ML − 1.5, DV − 1.5 mm). Next, two microscrews, used for ground and reference as well, were, respectively, fixed on the occipital bone above the cerebellum. (b) Recording environment and protocol. The mouse implanted with microelectrode recovered for 5-7 days after operation, the connector was connected to a multichannel EEG amplifier system, and the neural electrical signals were collected. EEG signal was collected in freely moving mice at 10: 00 am to 11: 00 am. It is noteworthy that signal acquisition had been carried out in their own cages. (c) Data acquisition. The original recording signal collected by the recording electrode is amplified 1000x by the amplifier. The analog signals were digitized at a sampling frequency rate of 1 kHz by a CED Micro1401 data acquisition system. A/D sampling rate was not only 128 Hz, and further, the signal was band-passed filter between 1 and 64 Hz. (d) Data analysis. Power spectral densities (PSDs) were generated every 30 s for each recording to calculate the amount of time spent at >6 Hz (time in high frequency). Signal processing ensured that integer values represented the dominant frequency (DF) in Hz for each 30 s epoch. The mean DF was calculated for each mouse from each DF in each 30 s epoch. The time in high frequency was calculated by summing the number of 30 s epochs with a DF > 6 Hz and dividing it by the total number of 30 s epochs (total time of the recording) for each mouse [7, 38].

### 2.8. Western Blotting

Following the EEG recording (a week later), the mice were killed by cervical dislocation and decapitation. The brains were rapidly placed into ice-cold phosphate-buffered saline (PBS) in a sterile dish and split into two parts. Half of the hippocampus from the exercised and sedentary mice were harvested for western blotting. The remaining tissues were prepared for cell-surface biotinylation assays, as described below.

For western blotting, the tissues were separately homogenized on ice in lysis buffer, comprising 10% sodium dodecyl sulfate (SDS), 10 mM Tris-HCl buffer (pH 7.4), 30% Triton-1000, 10 mM ethylenediaminetetraacetic acid (EDTA), protease inhibitor cocktail (Roche, Switzerland), and NaCl, using a homogenizer. The homogenates were centrifuged at 5,000 g for 10 min at 4°C. The protein was quantified using the bicinchoninic acid reagent (Sigma-Aldrich; Merck KGaA, Darmstadt, Germany) method. Equal amounts of the proteins were resolved by SDS-polyacrylamide gel electrophoresis (PAGE) on 4–12% gels, transferred to nitrocellulose membranes, and incubated with antibodies against Nav1.1*α* (1 : 800; cat. no. ASC-001; Alomone), Nav1.2 (1 : 200; cat. no. ASC-002; Alomone), Nav1.6 (1 : 500; cat. no. ab65166; Abcam), or Nav*β*2 (1 : 500; cat. no. ASC-007; Alomone). *β*-Actin (mouse monoclonal anti-*β*-actin; 1 : 800; Santa Cruz, Delaware, CA, USA) was used as a reference. The membranes were subsequently incubated with matched secondary antibodies at 20–25°C for 2 h. Horseradish peroxidase-conjugated antirabbit antibodies were used for Nav1.1*α*, Nav1.2, Nav1.6, and Nav*β*2 detection (1 : 2,500; cat. no. PI-1000; Vector Laboratories, Inc.), and a peroxidase-conjugated antimouse secondary antibody (1 : 3,000; cat. no. PI-2000; Vector Laboratories, Inc.) was used for *β*-actin detection. Enhanced chemiluminescence luminol reagent (Beyotime Institute of Biotechnology, Shanghai, China) was employed for protein quantification. A densitometric analysis of the target protein bands was conducted using a Bio-Rad Gel Imaging System (ChemiDoc™ XRS+; Bio-Rad Laboratories, Inc., Hercules, CA, USA) with Quantity One software v4.6.6 (Bio-Rad Laboratories, Inc.) for each group in order to quantify the protein expression levels.

### 2.9. Nav1.1*α* Cell-Surface Biotinylation Assay

The remaining hippocampal tissues were placed into ice-cold Krebs solution containing components as described previously [7] with modification (in mM): 120 NaCl, 4.5 KCl, 1.5 KH_2_PO_4_, 10 glucose, 1.5 MgSO_4_, 26 NaHCO_3_, and 1.5 CaCl_2_. Cell-surface biotinylation and detection of cell-surface Nav1.1*α* were performed as previously described [7, 38]. NeutrAvidin-agarose beads (Pierce, USA) were employed to pull down the biotinylated proteins, which were eluted by incubation with SDS-PAGE sample buffer at 37°C for 60 min and analyzed by SDS-PAGE followed by western blotting as described above. Biotinylated cell-surface proteins were bound to the beads for extracellular expression analysis, whereas the remaining lysate containing nonbiotinylated proteins was used for detection of the intracellular proteins.

### 2.10. Statistical Analysis

SPSS 19.0 for Windows covariance software package was used for the statistical analyses. The data are expressed as mean ± standard deviation (SD). The differences between the two groups were evaluated using Student's *t*-test. One-variable experiments with more than two groups were evaluated using analysis of variance (ANOVA) followed by Bonferroni's post hoc test. Two-way repeated-measures (RM) ANOVA followed by Tukey's test were employed for the MWM test analysis. The NOR data was analyzed using two-way ANOVA. EEG data were analyzed by one-way ANOVA, followed by Student's *t*-test for paired groups (two-tailed). *P* < 0.05 was considered significant.

## 3. Results

### 3.1. Exercise Improved Behavior Results

**Figure 1:**
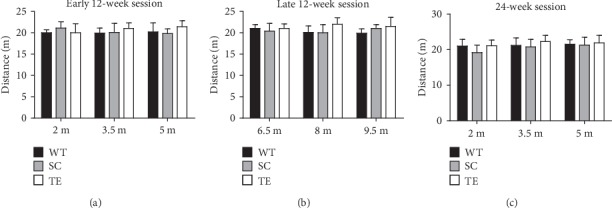
Effect of exercise on behavior in open field assessment. APP/PS1 mice in the early 12-week (a), late 12-week (b), and 24-week (c) groups showed no significant difference in horizontal or vertical movements compared with the WT control group. WT: wild type; SC: sedentary control; TE: treadmill exercise.

During the 10-minute open field assessment, the total distance showed no significant difference between the exercise groups and the age-matched sedentary controls for both APP/PS1 and WT mice (Figure 1, *F*-value, *P* value, and *df* were showed in the supplementary data [Supplementary-material supplementary-material-1]). Thus, the results indicated that gross locomotor and exploratory activity showed no significant difference between APP/PS1 mice and the WT controls with or without exercise.

The NOR and MWM tests were performed to determine whether treadmill exercise affected recognition memory and spatial memory deficiencies associated with AD development in APP/PS1 mice.

**Figure 2:**
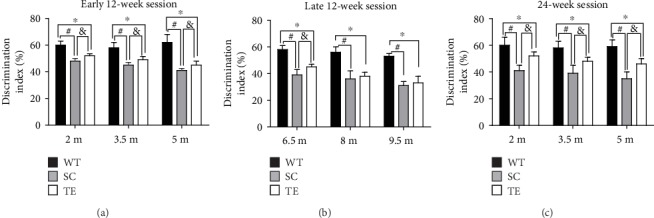
Treadmill exercise improved NOR task recognition memory in APP/PS1 mice. APP/PS1 mice in the early 12-week (a), late 12-week (b), and 24-week (c) groups showed significant differences in recognition memory impairment in the NOR task compared with the WT mice. Treadmill exercise for 12 and 24 weeks at least partially improved NOR memory. WT: wild type; SC: sedentary control; TE: treadmill exercise. ^∗^WT vs. TE, *P* < 0.05; ^#^WT vs. SC, *P* < 0.05; ^&^TE vs. SC, *P* < 0.05.

Compared to the WT mice, age-matched sedentary APP/PS1 mice had increased NOR task recognition memory deficiency (indicated by DI), which gradually increased with age (Figure 2). Following the early 12-week exercise regimen, the novel object exploration preference increased in APP/PS1 mice in all three age groups (2 m, 3.5 m, and 5 m) compared with the age-matched APP/PS1 sedentary controls (*P* < 0.05, Figure 2(a), *F*-value, *P* value, and *df* were showed in the supplementary data [Supplementary-material supplementary-material-1]). Following the late 12-week exercise regimen, the 6.5 m APP/PS1 mice showed a significant increase in novel object exploration preference compared to the age-matched APP/PS1 sedentary controls (*P* < 0.05, Figure 2(b), *F* value, *P* value, and *df* were in supplementary data [Supplementary-material supplementary-material-1]), but the 8 m and 9.5 m mice showed no significant difference compared with the age-matched sedentary controls (*P* > 0.05, Figure 2(b), *F*-value, and *df* were showed in the supplementary data [Supplementary-material supplementary-material-1]). Following the 24-week exercise regimen, the novel object exploration preference increased in APP/PS1 mice in all the three age groups (2 m, 3.5 m, and 5 m) compared with the age-matched APP/PS1 sedentary controls (*P* < 0.05, Figure 2(c), *F*-value, *P* value, and *df* were showed in the supplementary data [Supplementary-material supplementary-material-1]). The results indicated that exercised mice were protected from APP/PS1 mutation-induced impaired NOR task recognition memory, which deteriorated with age in APP/PS1 mice. The earlier the exercise treatment in APP/PS1 mice, the better the exercise-induced improvement in NOR task recognition memory. The results suggested that exercise is a promising therapeutic method for cognitive deficiency prevention rather than care for elderly individuals.

**Figure 3:**
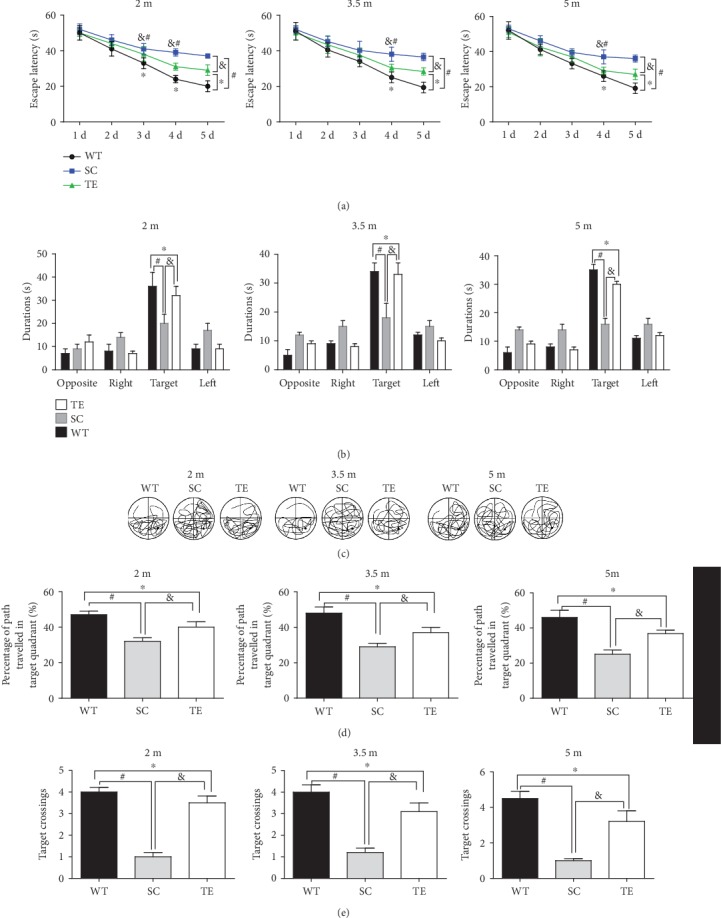
Early 12-week treadmill exercise improved spatial learning and memory in APP/PS1 mice. In the hidden platform test, the escape latencies of WT or APP/PS1 mice over 5 d were recorded (a). In the probe test, the percentage of time spent in the target quadrant (b), typical probe traces (c), percentage of path traveled in the target quadrant (d), and number of target crossings (e) for 2 m, 3.5 m, and 5 m WT and APP/PS1 mice with or without exercise and WT mice were compared. WT: wild type; SC: sedentary control; TE: treadmill exercise. ^∗^WT vs. TE, *P* < 0.05; ^#^WT vs. SC, *P* < 0.05; ^&^TE vs. SC, *P* < 0.05.

**Figure 4:**
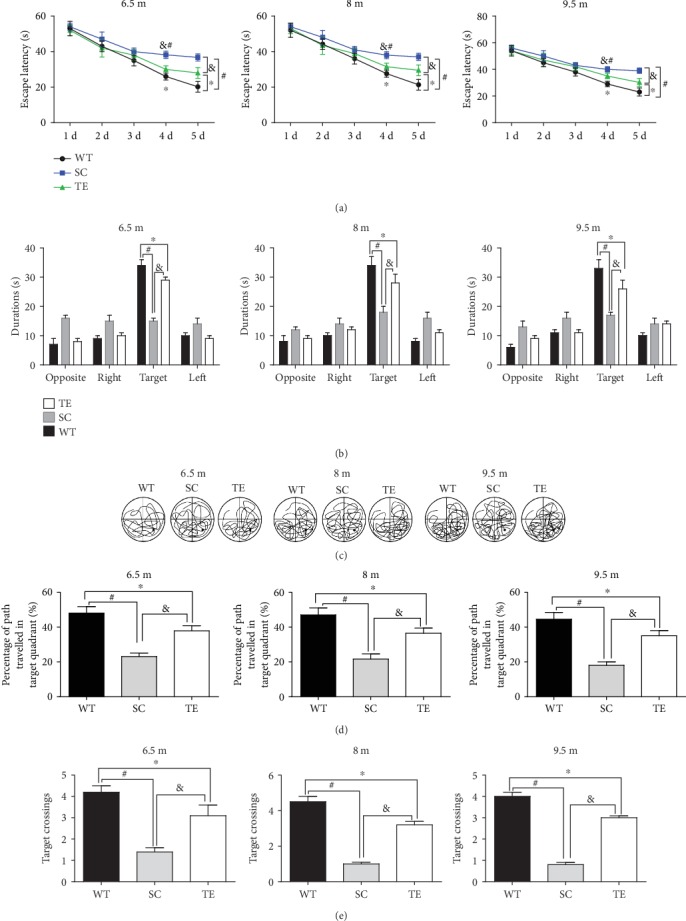
Late 12-week treadmill exercise improved spatial learning and memory in APP/PS1 mice. In the hidden platform test, the escape latencies of WT or APP/PS1 mice over 5 d were recorded (a). In the probe test, the percentage of time spent in the target quadrant (b), typical probe traces (c), percentage of path traveled in the target quadrant (d), and number of target crossings (e) for 6.5 m, 8 m, and 9.5 m WT and APP/PS1 mice with or without exercise and WT mice were compared. WT: wild type; SC: sedentary control; TE: treadmill exercise. ^∗^WT vs. TE, *P* < 0.05; ^#^WT vs. SC, *P* < 0.05; ^&^TE vs. SC, *P* < 0.05.

**Figure 5:**
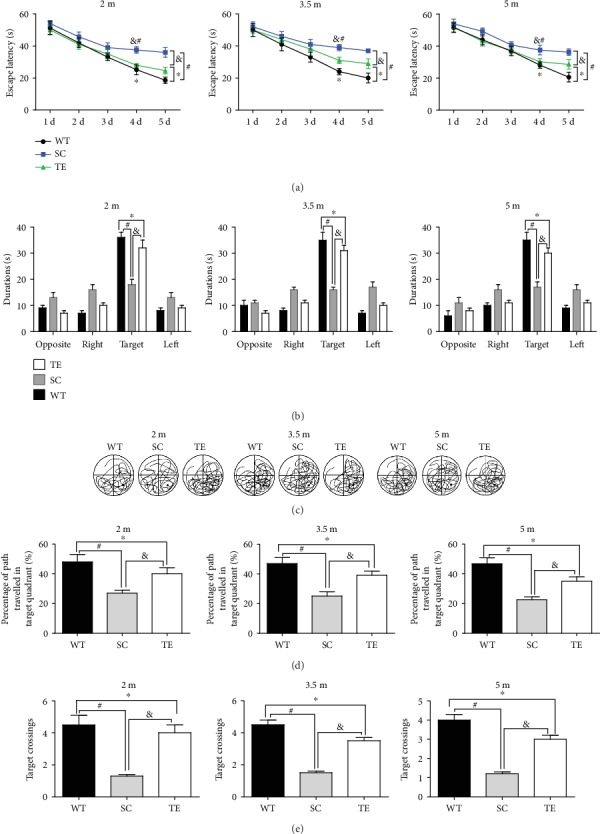
24-week treadmill exercise improved spatial learning and memory in APP/PS1 mice. In the hidden platform test, the escape latencies of WT or APP/PS1 mice over 5 d were recorded (a). In the probe test, the percentage of time spent in the target quadrant (b), typical probe traces (c), percentage of path traveled in the target quadrant (d), and number of target crossings (e) for 2 m, 3.5 m, and 5 m WT and APP/PS1 mice with or without exercise and WT mice were compared. WT: wild type; SC: sedentary control; TE: treadmill exercise. ^∗^WT vs. TE, *P* < 0.05; ^#^WT vs. SC, *P* < 0.05; ^&^TE vs. SC, *P* < 0.05.

Subsequently, the protection against APP/PS1 mutation-induced spatial memory deficits was verified using the MWM test. Two-way RM ANOVA revealed that the mean escape latency in the hidden platform test progressively decreased over time in all the groups (treadmill exercise groups, sedentary control groups, and WT control groups) within 60 seconds. The sedentary APP/PS1 mice had an increased escape latency compared to the age-matched WT or exercised APP/PS1 mice (Figures 3, 4, and 5). During the probe trial, two-way ANOVA with Tukey's test showed that 12- and 24-week exercised APP/PS1 mice had higher percentages of time in the target quadrant, percentages of their path in the target quadrant, and number of platform crossings (*P* < 0.05, Figures 3, 4, and 5, *F*-value, *P* value, and *df* were in supplementary data [Supplementary-material supplementary-material-1]) than age-matched APP/PS1 sedentary controls or WT mice. The preference for the target quadrant was not significantly different between 24-week exercised mice in the 2 m age group and age-matched sedentary APP/PS1 mice. Notably, there was no significant difference in speed observed between groups (supplementary data [Supplementary-material supplementary-material-1]). The data suggested that a long-term (24 weeks) treadmill exercise treatment at the early stage of AD is a promising strategy for protection against AD-induced spatial learning and memory deficits.

**Figure 6:**
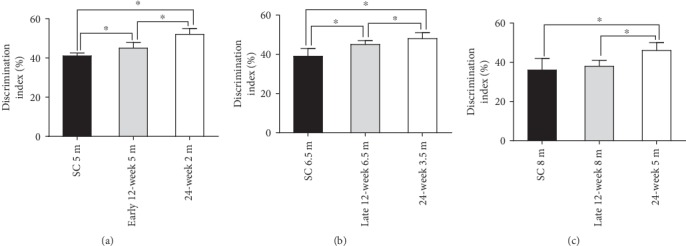
Effects on NOR task recognition memory of different exercise durations. Data from exercised APP/PS1 mice that were the same age at the end of the observation period are shown. (a) APP/PS1 mice aged 5 m in the early 12-week group and 2 m in the 24-week group (which were all the same age at the end of the observation period) were paired, while the sedentary control mice aged 5 m in the early 12-week group served as the negative controls. (b) APP/PS1 mice aged 6.5 m in the late 12-week group and 3.5 m in the 24-week group (which were all the same age at the end of the observation period) were paired, while the sedentary control mice aged 6.5 m in the late 12-week group served as the negative controls. (c) APP/PS1 mice aged 8 m in the late 12-week group and 5 m in the 24-week group (which were all the same age at the end of the observation period) were paired, while the sedentary control mice aged 8 m in the late 12-week group served as the negative controls. SC: sedentary control; TE: treadmill exercise. ^∗^*P* < 0.05.

**Figure 7:**
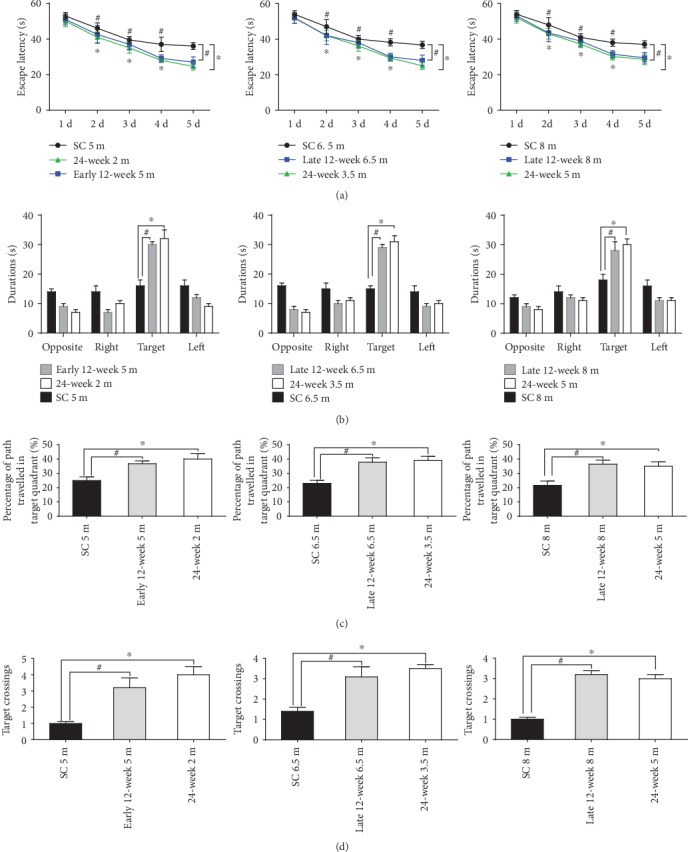
Effects on MWM spatial memory of different exercise durations. In the hidden platform test, the escape latencies (a) of APP/PS1 mice that were the same age at the end of the observation period were collected and analyzed. In the probe test, durations in the target quadrant (b), percentage of path traveled in the target quadrant (c), and number of target crossings (d) were compared. SC: sedentary control; TE: treadmill exercise. ^#^SC vs. 12-week exercise group, *P* < 0.05; ^∗^SC vs. 24-week exercise group, *P* < 0.05.

Additionally, we compared the effects of treadmill exercise with different durations on behavior performance between exercised APP/PS1 mice that were the same age at the end of the observation period (Figures 6 and 7). In the NOR task, the novel object exploration preference increased in the 24-week exercised APP/PS1 mice compared to the age-matched 12-week exercised APP/PS1 mice at the end of the observation period (Figure 6). The results indicated that 24-week exercised mice had decreased NOR task recognition memory deficiency (indicated by DI) compared to the 12-week exercised mice at the end of the study. In contrast, 8 m mice in the late 12-week exercise APP/PS1 group did not even differ significantly from the age-matched sedentary controls. In the MWM test, the 24-week exercised APP/PS1 mice showed no significant difference compared to the age-matched 12-week exercised APP/PS1 mice (Figure 7).

### 3.2. Treadmill Exercise Redresses Neuronal Hyperexcitability and Regulated the Expression of Nav

The EEG recordings in 2 m and 3.5 m sedentary APP/PS1 mice in the early 12-week group showed no significant difference compared to WT mice. Abnormal EEG patterns with spike-wave discharges (SWDs) were found in sedentary APP/PS1 mice in the late 12- and 24-week groups (in all age groups) and in 5 m sedentary APP/PS1 mice in the early 12-week group, compared to WT mice. Bilaterally synchronous generalized SWDs in EEG reflect high synchronized oscillations in the corticothalamo-cortical network [40]. The period of the spike wave is usually 83-200 ms, and the amplitude is above 100 *μ*V. The representative EEG recordings in these APP/PS1 mice exhibited longer durations of high-frequency brain activity, with some obvious spikes, which is consistent with previous reports [7, 38].

**Figure 8:**
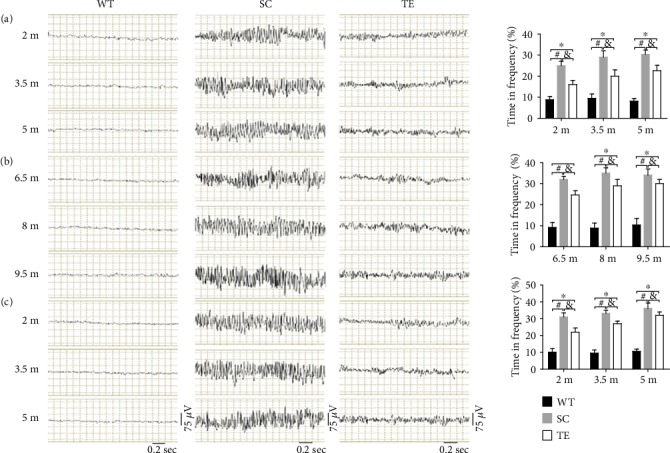
Treadmill exercise reversed hyperexcitability in APP/PS1 mice. EEG recordings showed increased brain activity amplitudes and frequencies of sedentary APP/PS1 mice in early 12-week (a), late 12-week (b), and 24-week (c) groups, which was partially reversed in the APP/PS1 mice treated with 12- and 24-week treadmill exercises. The percentage of time at high frequency (>6 Hz) was also recorded, analyzed, and indicated by histograms; exercised APP/PS1 mice exhibited shorter durations of high-frequency activity than the sedentary mice, but the levels did not reach WT levels. WT: wild type; SC: sedentary control; TE: treadmill exercise. ^∗^WT vs. TE, *P* < 0.05; ^#^WT vs. SC, *P* < 0.05; ^&^TE vs. SC, *P* < 0.05.

These abnormal EEG patterns were at least partially mitigated following 12- or 24-week exercise in APP/PS1 mice (Figure 8, *F*-value, *P* value, and *df* were showed in the supplementary data [Supplementary-material supplementary-material-1]). The 2 m APP/PS1 mice in the 24-week group exhibited comparatively normal neuronal activity without epileptiform discharges, with no significant difference compared to WT mice. However, the exercised-induced recovery in the other exercise groups did not reach WT levels. The percentages of time in frequency increased significantly in APP/PS1 mice without exercise, when compared with the age-matched WT mice or treadmill exercised mice, respectively (Figures 8(a), 8(b), and 8(c), *F*-value, *P* value, and *df* were showed in the supplementary data [Supplementary-material supplementary-material-1]). These results suggested that long-term treadmill exercise treatment in early-stage AD is a promising strategy.

APP/PS1 mutation-induced aberrant hyperexcitability is probably associated with Nav regulation. Nav1.1a, Nav1.2, and Nav1.6 are the major sodium channel subtypes on excitatory neurons, and they are responsible for neuronal excitability. In previous research, excessive Nav*β*2 cleavage induced by BACE1 overexpression in an AD mouse model retained intracellular levels and reduced surface levels of Nav1.1*α*, decreasing action potential propagation and neuronal activity [15, 23]. Therefore, we investigated whether treadmill exercise recovered APP/PS1 mutation-induced aberrant neuronal excitability by modulating the expression of sodium channels.

**Figure 9:**
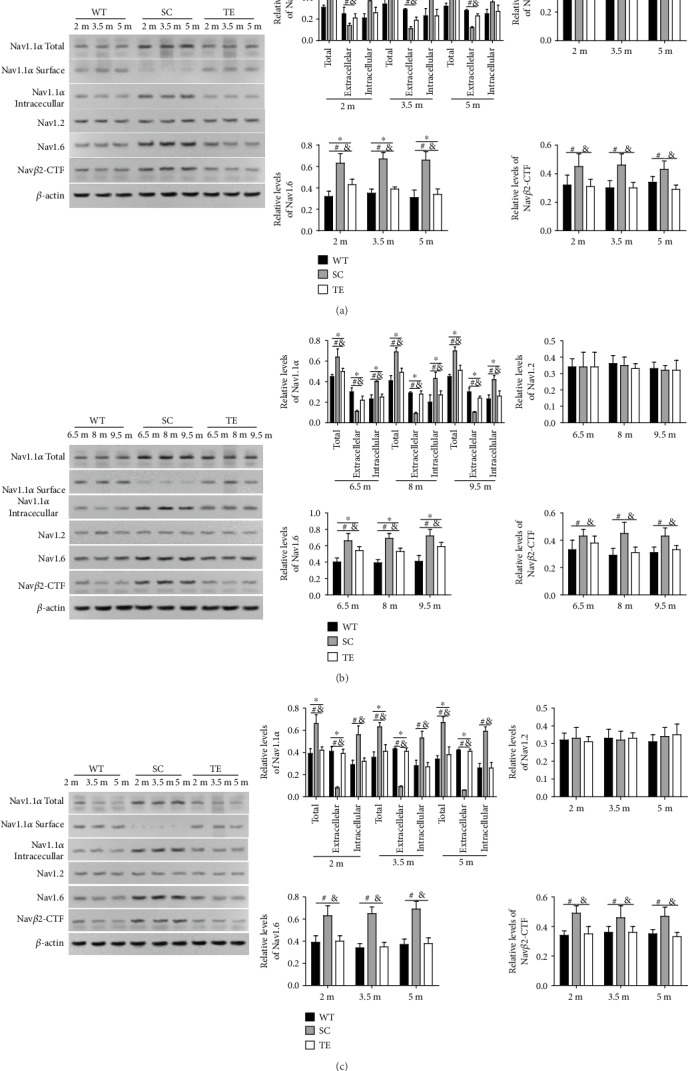
Treadmill exercise redressed aberrant sodium channel expression in APP/PS1 mice. Hippocampal Nav expression levels were detected in mice in the early 12-week (a), late 12-week (b), and 24-week (c) groups. Aberrant Nav expression was observed in APP/PS1 mice with different ages, which were altered following 12- or 24-week treadmill exercise. WT: wild type; SC: sedentary control; TE: treadmill exercise. ^∗^WT vs. TE, *P* < 0.05; ^#^WT vs. SC, *P* < 0.05; ^&^TE vs. SC, *P* < 0.05.

The levels of Nav1.1*α* (total, extracellular, and intracellular), Nav1.2, Nav1.6, and Nav*β*2-C-terminal fragment (CTF) showed different changes in WT mice with different ages, while APP/PS1 mutation altered the protein expression of these Nav family members (Figure 9).

Among the Nav family members, increased hippocampal Nav*β*2-CTF was observed in the sedentary APP/PS1 mice in early 12-week exercised mice, late 12-week exercised mice, and 24-week exercised mice (*P* < 0.05, Figure 9, *F*-value, *P* value, and *df* were showed in the supplementary data [Supplementary-material supplementary-material-1]). Further, decreased Nav*β*2-CTF was detected in the hippocampus and cerebral cortex of the 12-week and 24-week sedentary APP/PS1 mice. In fact, Nav*β*2-CTF expression in the 12- and 24-week exercised APP/PS1 mice was not significantly different compared to the age-matched WT mice (*P* < 0.05, Figure 9, *F*-value, *P* value, and *df* were showed in the supplementary data [Supplementary-material supplementary-material-1]). As indicated by the cell-surface biotinylation assays, the total and intracellular levels of Nav1.1*α* increased, while extracellular (cell-surface) Nav1.1*α* was nearly absent in the hippocampal regions of sedentary APP/PS1 mice. Nav1.6 increased with age in the sedentary APP/PS1 mice, and it significantly differed from the age-matched WT mice (*P* < 0.05, Figure 9, *F*-value, *P* value, and *df* were showed in the supplementary data [Supplementary-material supplementary-material-1]).

Both early and late 12- and 24-week exercises effectively reduced the total and cell-surface Nav1.1*α*, Nav*β*2-CTF, and Nav1.6 and restored the intracellular Nav1.1*α* levels in APP/PS1 mice compared to age-matched sedentary APP/PS1 mice (*P* < 0.05, Figure 9, *F*-value, *P* value, and *df* were showed in the supplementary data [Supplementary-material supplementary-material-1]). Additionally, Nav1.6 expression in the 24-week exercised APP/PS1 mice was not significantly different compared to the age-matched WT mice (*P* > 0.05, Figure 9, *F*-value, *P* value, and *df* were showed in the supplementary data [Supplementary-material supplementary-material-1]). There were no significant alterations in Nav1.2 expression in APP/PS1 and WT mice throughout the study, regardless of exercise (data not shown).

## 4. Discussion

In this study, we demonstrated that 12- and 24-week treadmill exercise regimens in APP/PS1 mice improved short-term recognition memory (indicated by the NOR task) and spatial memory (indicated by the MWM test), as well as restoring neuronal excitability (indicated by EEG recordings). Consistent with the restored neuronal excitability, exercise also partially redressed the aberrant hippocampal expression and localization of Nav proteins, including Nav1.1a, Nav1.6, and Nav*β*2. This suggested that long-term treadmill exercise improved APP/PS1 mutation-induced cognitive defects by partially reversing aberrant neuronal activity and/or seizures, which was probably associated with the recovery of hippocampal Nav.

Physical exercise is critical in maintaining brain health and cognitive performance in older adults [9]. Although the optimal exercise dosage (intensity and duration) and type of exercise remain unclear, positive relationships between a higher dosage of exercise and cognitive health have been reported in older adults [9]. Research using animal models also indicates the promising effects of exercise [12]. As a promising type of exercise, treadmill exercise improved cognitive decline in an AD mouse model, and this exercise has therapeutic potential for the prevention or treatment of AD. A previous study demonstrated that 12-week treadmill exercise can effectively prevent decreased hippocampal-related cognitive function and reduce A*β* deposits in early-stage AD, possibly by modulating microglia-mediated neuroinflammation and oxidative stress [41]. Other research showed that 5-month (long-term) treadmill exercise in APP/PS1 transgenic mice was sufficient to inhibit AD-like neuropathology progression and improve hippocampal cognitive deficits by enhancing long-term potentiation (LTP), suppressing both A*β*-42 and neuronal death pathways, and inducing APP processing to reduce A*β* deposition [34, 35, 42]. We found that 12- and 24-week treadmill exercises with moderate intensity improved cognitive performance in APP/PS1 transgenic mice, which concurs with previous reports [9, 34]. Interestingly, we also observed that mice subjected to a longer duration (24 weeks) of treadmill exercise (in all three age groups) showed increased novel object exploration preference compared to age-matched 12-week exercised APP/PS1 mice. In contrast, the late 12-week exercise regimen failed to improve NOR task recognition memory in the older (8 m and 9.5 m) mice. However, no significant differences were found in MWM performance between the 24-week exercised APP/PS1 mice and the age-matched 12-week exercised APP/PS1 mice. These results suggest that 24-week treadmill exercise has an advantage over late 12-week treadmill exercise in improving recognition memory in APP/PS1 mice, but not in improving spatial memory. Our findings support the need for longer-duration exercise interventions at the early stage of dementia to bring about improvement in short-term recognition memory, although further research is needed in this area. Both 12- and 24-week exercises had positive effects on hippocampus-dependent spatial memory.

Paralleled to cognitive improvements in AD mice, we also revealed that treadmill exercise partially redressed neural hyperexcitability and altered Nav distribution and/or expression in the hippocampus. The 12- or 24-week exercise regimens ameliorated the abnormal expression and localization of Nav1.1a, Nav1.6, and Nav*β*2. Moreover, 24-week exercise completely reversed Nav1.6 expression, with no significant difference compared to the age-matched WT mice. We proposed that treadmill exercise-induced improvement in memory and neural activity was associated with altering the numbers and distribution of hippocampal Nav in the AD mouse model.

Negative associations between increased hippocampal activation and cognitive health or AD development have been demonstrated in humans and animal models [5, 43], though the mechanisms are not well understood. Exercise has been shown to reduce hippocampal network hyperexcitability and protect against seizure susceptibility [12]. Some authors suggest that the mechanisms by which exercise serves as a cognitive enhancer against hippocampal hyperactivity is associated with a brain-derived neurotrophic factor- (BDNF-) dependent increase in the growth of neurons [11]. Other authors suggest that the protective effects of exercise against seizures are at least partially mediated by the regulation of neural excitability in a process involving galanin [44]. Considering the importance of Nav in the generation and maintenance of neural excitability, we focused on the numbers and distribution of the sodium channels.

Nav are necessary components required to generate and propagate action potential depolarization and firing frequency, so they play a crucial role in excitable cells, especially in neurons [14, 45, 46]. In this study, the results revealed that 12- or 24-week treadmill exercise partially redressed the disturbed cellular distribution of Nav1.1*α*, aberrantly increased Nav*β*2 cleavage, as well as the increased Nav1.6 expression in the AD mice. The results indicate that exercise improved memory, and neural activity is associated with the altering of the numbers and distribution of hippocampal Nav in APP/PS1 mice. Evidence has demonstrated that Nav subunits are involved in AD progression-related neural hyperexcitability and cognitive deficits. We proposed that treadmill exercise-induced improvement in memory and neural activity was associated with altering the numbers and distribution of hippocampal Nav in the AD mouse model. The dysregulation of Nav1.1*α* levels and aberrant cleavage of Nav*β*2 were contributed to the BACE1 upregulation in cortical neurons, abnormal EEG activity, and cognitive deficits in AD mice [7, 46]. Increased SCN2B (encode gene of Nav*β*2) expression in the hippocampus was associated with cognitive deficits in the senescence-accelerated P8 mice [32], while Nav*β*2 knockdown reversed the APP/PS1 mutation-induced deficiency in amyloid *β* (A*β*) degradation by regulating NEP in APP/PS1 mouse-derived neurons [47], preserved neurons, redressed Nav1.1*α* distributions, and improved spatial cognition by partially decreasing pathological amyloidogenic APP processing in aged APP/PS1 mice [38]. A*β*1-42 treatment of cultured hippocampal neurons induced an upregulation of the Nav and Nav1.6 expression, as well as increasing neural excitability [48]. Accordingly, we propose that Nav*β*2 and/or Nav1.6 may play a main role in exercise-induced rescuing neuronal excitability in this study. Exercise-induced recovery of Nav*β*2 cleavage is responsible for the normalization of Nav1.1*α* due to the transcription regulated effects of the intracellular domain fragment of Nav*β*2 (*β*2-ICD) on Nav1.1*α* mRNA [7, 46]. A*β*1-42 treatment of cultured hippocampal neurons induced an upregulation of the Nav and Nav1.6 expression, as well as increasing neural excitability [48]. Treatment with a voltage-dependent sodium channel inhibitor decreased APP cleavage by BACE1 [3], reversing the synaptic disorder and cognitive dysfunction in APP transgenic mice [43, 49, 50]. These results suggest that AD-related factors, such as A*β*, play a significant role in regulating neuronal activity in specific types of neurons as well as in wider neuronal networks, and A*β* and sodium channels have a certain relationship [48]. Since the enhanced degradation of A*β* induced by Nav*β*2 knockdown in APP/PS1 mouse-derived neurons, it is possible that treadmill exercise-induced redressing in elevated Nav*β*2 cleavage may attenuate A*β*-regulated neural excitability by decreased APP amyloid processing [47]. However, further investigation is necessary to reveal the mechanisms underlying. A long-term treadmill exercise regimen, for about 24 weeks, intervening initially at the preclinical stage, is a promising therapeutic approach due to the increased improvement in memory and Nav expression (especially Nav1.6) in the AD mice subjected to the 24-ewek exercise regimen.

There was no differential expression of Nav1.2 in the 12- and 24-week exercise groups. Additionally, the limitation of this study is that we did not carry out experiments to evaluate the pathological changes of AD model before or after treadmill exercise treatment, such as Aß plagues or expression of Tau. In sum, these results in our study indicate that treadmill exercise protects against cognition dysfunction and hippocampal hyperexcitability induced by AD in a manner independent of Nav1.2 regulation.

This study supplied evidence that specific exercise durations have differential effects on cognitive performance in AD models.

## Figures and Tables

**Table 1:** Grouping of APP/PS1 mice for various lab and experimental procedures.

Lab/behavioral investigations			Behavior evaluation (OF/NOR/MWM)	EEG recording	Nav expression analysis (Western blotting/cell surface biotinylation assay)
Grouping of APP/PS1 mice			Number of mice		
Early 12-week	Sedentary control	2 m	11	5	11
		3.5 m	11	5	11
		5 m	11	5	11
	Treadmill exercise	2 m	11	5	11
		3.5 m	10^∗^	5	10^∗^
		5 m	9^∗^	5	9^∗^

Late 12-week	Sedentary control	6.5 m	10^∗^	4^∗^	10^∗^
		8 m	11	5	11
		9.5 m	11	5	11
	Treadmill exercise	6.5 m	10^∗^	5	10^∗^
		8 m	10^∗^	5	10^∗^
		9.5 m	8^∗^	3^∗^	8^∗^

24-week	Sedentary control	2 m	11	5	11
		3.5 m	11	5	11
		5 m	11	5	11
	Treadmill exercise	2 m	11	5	11
		3.5 m	10^∗^	5	10^∗^
		5 m	9^∗^	4^∗^	9^∗^

^∗^Mice that died in this group were dropped out. The number of mice shows the actual number included in the analysis at the end of the experimental procedures; 2 m, 2-month-old; 3.5 m, 3.5-month-old; 5 m, 5-month-old; 6.5 m, 6.5-month-old; 8 m, 8-month-old; 9.5 m, 9.5-month-old. The age of the mice in the table corresponds to the age at the beginning of the study. OF: open field; NOR: novel object recognition task; MWM: Morris water maze test; EEG: electroencephalogram.

**Table 2:** Grouping of WT mice for various lab and experimental procedures.

Lab/behavioral investigation		Behavior evaluation (OF/NOR/MWM)	EEG recording	Nav expression analysis (Western blotting/cell-surface biotinylation assay)
Grouping of WT mice		Number of mice		
Early 12-week WT control	2 m	11	5	11
	3.5 m	11	5	11
	5 m	11	5	11

Late 12-week WT control	6.5 m	11	5	11
	8 m	11	5	11
	9.5 m	11	5	11

24-week WT control	2 m	11	5	11
	3.5 m	11	5	11
	5 m	11	5	11

2 m, 2-month-old; 3.5 m, 3.5-month-old; 5 m, 5-month-old; 6.5 m, 6.5-month-old; 8 m, 8-month-old; 9.5 m, 9.5-month-old. The age of the mice in the table corresponds to the age at the beginning of the study. The mice in the early 12-week WT control groups survived for 12 weeks and were used for behavior evaluation and then sacrificed for the Nav protein analysis, as in the other groups. OF; open field; NOR: novel object recognition task; MWM: Morris water maze test; EEG: electroencephalogram.

**Table 3:** The total object exploration time in NOR test.

Grouping of APP/PS1 mice			Exploration time (%)		Total exploration time for both objects (min)
			Familiar	Novel	
Early 12-week	WT	2 m	40 ± 2.8	60 ± 3.4	7.3 ± 1.3
		3.5 m	42 ± 3.9	58 ± 4.1	8.5 ± 1.1
		5 m	38 ± 4.8	62 ± 4.1	7.7 ± 2.9
	Sedentary control	2 m	52 ± 3.4	48 ± 2.1	8.3 ± 1.9
		3.5 m	55 ± 2.8	45 ± 2.6	7.9 ± 2.8
		5 m	59 ± 3.7	41 ± 1.5	6.8 ± 2.4
	Treadmill exercise	2 m	58 ± 2.5	52 ± 1.5	6.7 ± 2.2
		3.5 m	51 ± 1.9	49 ± 2.5	7.4 ± 1.1
		5 m	55 ± 4.2	45 ± 3.2	8.3 ± 2.1

Late 12-week	WT	6.5 m	42 ± 3.6	58 ± 3.2	8.8 ± 1.2
		8 m	44 ± 4.9	56 ± 4.1	9.1 ± 3.2
		9.5 m	47 ± 3.1	53 ± 2.2	7.6 ± 2.7
	Sedentary control	6.5 m	61 ± 5.1	39 ± 4.8	7.7 ± 2.4
		8 m	64 ± 5.9	36 ± 6.2	8.4 ± 3.4
		9.5 m	69 ± 8.2	31 ± 3.7	7.6 ± 2.4
	Treadmill exercise	6.5 m	55 ± 3.6	45 ± 2.3	7.2 ± 2.8
		8 m	62 ± 5.4	38 ± 3.4	8.1 ± 3.5
		9.5 m	67 ± 6.9	33 ± 5.2	8.4 ± 2.6

24-week	WT	2 m	40 ± 2.7	60 ± 6.4	8.3 ± 2.6
		3.5 m	42 ± 3.1	58 ± 5.1	7.8 ± 1.9
		5 m	41 ± 3.3	59 ± 5.6	8.9 ± 3.1
	Sedentary control	2 m	59 ± 5.9	41 ± 4.3	7.7 ± 3.4
		3.5 m	61 ± 8.1	39 ± 6.1	8.2 ± 4.1
		5 m	65 ± 7.2	35 ± 5.7	7.5 ± 2.7
	Treadmill exercise	2 m	48 ± 5.1	52 ± 3.2	8.6 ± 2.7
		3.5 m	52 ± 3.7	48 ± 3.6	7.9 ± 1.8
		5 m	54 ± 6.6	46 ± 4.7	7.3 ± 2.8

2 m, 2-month-old; 3.5 m, 3.5-month-old; 5 m, 5-month-old; 6.5 m, 6.5-month-old; 8 m, 8-month-old; 9.5 m, 9.5-month-old. The age of the mice in the table corresponds to the age at the beginning of the study.

## Data Availability

The data used to support the findings of this study are included within the supplementary data.

## Supplementary Materials

Supplementary Materials The supplementary data contents the following two aspects: part 1 are the full ANOVA stats (with *P* value, degrees of freedom, and *F*-values) for each of the measured parameter in Section 3; part 2 in supplementary data are the ANOVA analysis (with *P* value, degrees of freedom, and *F*-values) for the swimming speed of mice in MWM test.
