# Supplementary material for: Exercise-Induced Cognitive Improvement Is Associated with Sodium Channel-Mediated Excitability in APP/PS1 Mice
Source: Neural Plast. 2020 Mar 18;2020:9132720. doi: 10.1155/2020/9132720 (PMC7103997; doi:10.1155/2020/9132720)
Supplement: Supplementary Materials — The supplementary data contents the following two aspects: part 1 are the full ANOVA stats (with P value, degrees of freedom, and F-values) for each of the measured parameter in Section 3; part 2 in supplementary data are the ANOVA analysis (with P value, degrees of freedom, and F-values) for the swimming speed of mice in MWM test. [file 9132720.f1.docx]

**Exercise-Induced Cognitive Improvement is Associated with Sodium Channel-Mediated Excitability in APP/PS1 Mice**

**Supplementary data**

The Supplementary data contents the following two aspects: part1 are the full ANOVA stats (with P value, degrees of freedom and F-values) for each of the measured parameter in the Results of this study; Part 2 in Supplementary data are the ANOVA analysis (with P value, degrees of freedom and F-values) for the swimming speed of mice in MWM test.

**Part 1. The full ANOVA stats (with degrees of freedom, P value and F-values) for each of the measured parameter in the Results.**

**Figure 1.**

The ANOVA analysis in open field assessment: early 12-week, p=0.378, p> 0.05, F-value =1.392, df = 97; late 12-week, p=0.47, p> 0.05, F-value =2.07, df =94; 24-week, p=0.73, p> 0.05, F-value =1.22, df = 97.

**Figure 2.**

Following the early 12-week exercise regimen, the novel object exploration preference increased in APP/PS1 mice in all three age groups (2m, 3.5m and 5m) compared with the age-matched APP/PS1 sedentary controls (2m: p=0.012, F-value =20.43; 3.5m: p=0.018, F-value =19.63; 5m: p=0.009, F-value =23.56; df=97, *p*<0.05, **Figure 2A**). Following the late 12-week exercise regimen, the 6.5m APP/PS1 mice showed a significant increase in novel object exploration preference compared to the age-matched APP/PS1 sedentary controls (p=0.02, F-value =25.82, df = 94, *p*<0.05, **Figure 2B**), but the 8m and 9.5m mice showed no significant difference compared with the age-matched sedentary controls (8m: p=0.65, F-value =1.01; 9.5m: p=0.77, F-value =1.2, df = 94; *p*>0.05, **Figure 2B**). Following the 24-week exercise regimen, the novel object exploration preference increased in APP/PS1 mice in all three age groups (2m, 3.5m and 5m) compared with the age-matched APP/PS1 sedentary controls (2m: p=0.000, F-value =48.21; 3.5m: p=0.001, F-value =29.01; 5m: p=0.000, F-value =31.02; df =97; *p*<0.05, **Figure 2C**).

**Figures 3, 4 and 5.**

The sedentary APP/PS1 mice had an increased escape latency compared to the age-matched WT or exercised APP/PS1 mice (early 12-week, df = 97: 2m, SC vs WT, p=0.000, F-value =43.67; SC vs TE: p=0.003, F-value =23.09; TE vs WT: p=0.009, F-value =18.24; 3.5m, SC vs WT, p=0.000, F-value =63.12; SC vs TE: p=0.01, F-value =18.03; TE vs WT: p=0.017, F-value =12.84; 5m, SC vs WT, p=0.002, F-value =33.43; SC vs TE: p=0.013, F-value =24.19; TE vs WT: p=0.032, F-value =13.43; Late 12-week, df = 94: 6.5m, SC vs WT, p=0.001, F-value =40.12; SC vs TE: p=0.009, F-value =18.92; TE vs WT: p=0.01, F-value =9.03; 8m, SC vs WT, p=0.000, F-value =29.03; SC vs TE: p=0.008, F-value =21.08; TE vs WT: p=0.013, F-value =19.04; 9.5m, SC vs WT, p=0.002, F-value =28.93; SC vs TE: p=0.018, F-value =19.05; TE vs WT: p=0.021, F-value =17.03; 24-week, df = 97: 2m, SC vs WT, p=0.000, F-value =43.09; SC vs TE: p=0.001, F-value =30.93; TE vs WT: p=0.015, F-value =10.93; 3.5m, SC vs WT, p=0.000, F-value =49.09; SC vs TE: p=0.002 , F-value =27.48; TE vs WT: p=0.022, F-value =17.03; 5m, SC vs WT, p=0.001, F-value =37.05; SC vs TE: p=0.019, F-value =24.08; TE vs WT: p=0.02, F-value =20.98; *p*<0.05; **Figures 3, 4 and 5**). During the probe trial, two-way ANOVA with Tukey’s test showed that 12- and 24-week exercised APP/PS1 mice had higher percentages of time in the target quadrant (early 12-week, df = 97: 2m, SC vs WT, p=0.001, F-value =33.98; SC vs TE: p=0.0028, F-value =20.97; TE vs WT: p=0.007, F-value =15.09; 3.5m, SC vs WT, p=0.001, F-value =38.12; SC vs TE: p=0.009, F-value =20.98; TE vs WT: p=0.024, F-value =16.34; 5m, SC vs WT, p=0.000, F-value =43.98; SC vs TE: p=0.027, F-value =22.09; TE vs WT: p=0.036, F-value =10.83; Late 12-week, df = 94: 6.5m, SC vs WT, p=0.0016, F-value =37.94; SC vs TE: p=0.0034, F-value =17.92; TE vs WT: p=0.024, F-value =8.09; 8m, SC vs WT, p=0.0003, F-value =30.98; SC vs TE: p=0.001, F-value =22.87; TE vs WT: p=0.027, F-value =13.65; 9.5m, SC vs WT, p=0.001, F-value =31.83; SC vs TE: p=0.007, F-value =23.09; TE vs WT: p=0.018, F-value =11.83; 24-week, df = 97: 2m, SC vs WT, p=0.001, F-value =38.33; SC vs TE: p=0.003, F-value =25.09; TE vs WT: p=0.021, F-value =9.04; 3.5m, SC vs WT, p=0.000, F-value =38.61; SC vs TE: p=0.0018 , F-value =26.04; TE vs WT: p=0.032, F-value =12.8; 5m, SC vs WT, p=0.000, F-value =48.27; SC vs TE: p=0.009, F-value =30.76; TE vs WT: p=0.034, F-value =17.64; *p*<0.05, **Figures 3, 4 and 5**), percentages of their path in the target quadrant (early 12-week, df = 97: 2m, SC vs WT, p=0.007, F-value =24.09; SC vs TE: p=0.01, F-value =13.98; TE vs WT: p=0.009, F-value =16.88; 3.5m, SC vs WT, p=0.0045, F-value =26.91; SC vs TE: p=0.029, F-value =13.05; TE vs WT: p=0.037, F-value =9.33; 5m, SC vs WT, p=0.001, F-value =26.09; SC vs TE: p=0.019, F-value =19.53; TE vs WT: p=0.027, F-value =11.53; Late 12-week, df = 94: 6.5m, SC vs WT, p=0.000, F-value =42.65; SC vs TE: p=0.005, F-value =22.49; TE vs WT: p=0.019, F-value =11.77; 8m, SC vs WT, p=0.000, F-value =48.03; SC vs TE: p=0.003, F-value =20.98; TE vs WT: p=0.013, F-value =16.32; 9.5m, SC vs WT, p=0.0014, F-value =41.87; SC vs TE: p=0.009, F-value =25.46; TE vs WT: p=0.029, F-value =10.63; 24-week, df = 97: 2m, SC vs WT, p=0.003, F-value =28.43; SC vs TE: p=0.013, F-value =18.38; TE vs WT: p=0.018, F-value =10.63; 3.5m, SC vs WT, p=0.002, F-value =25.09; SC vs TE: p=0.006, F-value =16.35; TE vs WT: p=0.021, F-value =9.53; 5m, SC vs WT, p=0.001, F-value =33.81; SC vs TE: p=0.008, F-value =25.9; TE vs WT: p=0.02, F-value =10.53; *p*<0.05, **Figures 3, 4 and 5**), and number of platform crossings (early 12-week, df = 97: 2m, SC vs WT, p=0.000, F-value =45.04; SC vs TE: p=0.0013, F-value =35.85; TE vs WT: p=0.02, F-value =7.03; 3.5m, SC vs WT, p=0.0013, F-value =39.36; SC vs TE: p=0.004, F-value =24.94,; TE vs WT: p=0.031, F-value =12.82; 5m, SC vs WT, p=0.000, F-value =47.92; SC vs TE: p=0.003, F-value =32.74; TE vs WT: p=0.017, F-value =23.11; Late 12-week, df = 94: 6.5m, SC vs WT, p=0.0026, F-value =30.34; SC vs TE: p=0.005, F-value =23.84; TE vs WT: p=0.017, F-value =10.83; 8m, SC vs WT, p=0.000, F-value =43.93; SC vs TE: p=0.0018, F-value =27.92; TE vs WT: p=0.019, F-value =14.96; 9.5m, SC vs WT, p=0.000, F-value =42.99; SC vs TE: p=0.0042, F-value =21.09; TE vs WT: p=0.029, F-value =12.17; 24-week, df = 97: 2m, SC vs WT, p=0.0009, F-value =40.39; SC vs TE: p=0.0013, F-value =32.79; TE vs WT: p=0.038, F-value =7.17; 3.5m, SC vs WT, p=0.003, F-value =27.87; SC vs TE: p=0.0023, F-value =20.98; TE vs WT: p=0.039, F-value =10.87; 5m, SC vs WT, p=0.0011, F-value =33.88; SC vs TE: p=0.01, F-value =23.73; TE vs WT: p=0.026, F-value =12.29; *p*<0.05, **Figure 3, 4 and 5**) than age-matched APP/PS1 sedentary controls or WT mice.

**Figure 8.**

The percentages of time in frequency increased significantly in APP/PS1 mice without exercise, when compared with the age-matched WT mice or treadmill exercised mice, respectively (early 12-week, df = 46: 2m, SC vs WT, p=0.002, F-value =35.09; SC vs TE: p=0.009, F-value =28.03; TE vs WT: p=0.027, F-value =10.99; 3.5m, SC vs WT, p=0.000, F-value =45.05; SC vs TE: p=0.002, F-value =34.86; TE vs WT: p=0.005, F-value =29.66; 5m, SC vs WT, p=0.000, F-value =57.92; SC vs TE: p=0.001, F-value =40.19; TE vs WT: p=0.0018, F-value =37.38; Late 12-week, df = 45: 6.5m, SC vs WT, p=0.0001, F-value =38.47; SC vs TE: p=0.008, F-value =29.11; TE vs WT: p=0.011, F-value =14.92; 8m, SC vs WT, p=0.000, F-value =42.58; SC vs TE: p=0.0024, F-value =26.18; TE vs WT: p=0.03, F-value =11.63; 9.5m, SC vs WT, p=0.001, F-value =39.05; SC vs TE: p=0.0024, F-value =23.66; TE vs WT: p=0.031, F-value =9.83; 24-week, df =45: 2m, SC vs WT, p=0.000, F-value =35.85; SC vs TE: p=0.0027, F-value =29.11; TE vs WT: p=0.011, F-value =19.45; 3.5m, SC vs WT, p=0.001, F-value =30.87; SC vs TE: p=0.029 , F-value =11.93; TE vs WT: p=0.012, F-value =23.99; 5m, SC vs WT, p=0.000, F-value =34.09; SC vs TE: p=0.02, F-value =9.08; TE vs WT: p=0.006, F-value =23.87; *p*<0.05, **Figure 8A, B and C**).

**Figure 9.**

The results of the ANOVA analysis are as follows: early 12-week, df =97: 2m, SC vs WT, p=0.009, F-value =21.85; SC vs TE: p=0.01, F-value =19.14; TE vs WT: p=0.19, F-value =1.28; 3.5m, SC vs WT, p=0.0061, F-value =23.06; SC vs TE: p=0.0056, F-value =23.11,; TE vs WT: p=0.43, F-value =2.46; 5m, SC vs WT, p=0.028, F-value =17.67; SC vs TE: p=0.017, F-value =18.49; TE vs WT: p=0.57, F-value =2.98; Late 12-week, df = 94: 6.5m, SC vs WT, p=0.013, F-value =17.34; SC vs TE: p=0.022, F-value =13.84; TE vs WT: p=0.26, F-value =2.81; 8m, SC vs WT, p=0.0084, F-value =19.93; SC vs TE: p=0.009, F-value =17.92; TE vs WT: p=0.63, F-value =3.96; 9.5m, SC vs WT, p=0.011, F-value =16.99; SC vs TE: p=0.029, F-value =13.29; TE vs WT: p=0.48, F-value =1.17; 24-week, df = 97: 2m, SC vs WT, p=0.012, F-value =16.09; SC vs TE: p=0.02, F-value =12.25; TE vs WT: p=0.28, F-value =1.32; 3.5m, SC vs WT, p=0.019, F-value =10.37; SC vs TE: p=0.032, F-value =6.98; TE vs WT: p=0.39, F-value =1.43; 5m, SC vs WT, p=0.018, F-value =13.09; SC vs TE: p=0.011, F-value =15.17; TE vs WT: p=0.45, F-value =1.03; **Figure 9**). As indicated by the cell-surface biotinylation assays, total and intracellular levels of Nav1.1α increased, while extracellular (cell-surface) Nav1.1α was nearly absent in the hippocampal regions of sedentary APP/PS1 mice. Nav1.6 increased with age in the sedentary APP/PS1 mice, and it significantly differed from the age-matched WT mice. Both early and late 12- and 24-week exercise effectively reduced the total and cell-surface Nav1.1α, Navβ2-CTF, and Nav1.6, and restored the intracellular Nav1.1α levels in APP/PS1 mice compared to age-matched sedentary APP/PS1 mice. Additionally, Nav1.6 expression in the 24-week exercised APP/PS1 mice was not significantly different compared to the age-matched WT mice. The results of the ANOVA analysis are described as follows: Nav1.6, early 12-week, df =97: 2m, SC vs WT, p=0.001, F-value =25.87; SC vs TE: p=0.009, F-value =19.36; TE vs WT: p=0.027, F-value =13.84; 3.5m, SC vs WT, p=0.0021, F-value =23.28; SC vs TE: p=0.004, F-value =20.18; TE vs WT: p=0.02, F-value =9.47; 5m, SC vs WT, p=0.0009, F-value =27.66; SC vs TE: p=0.001, F-value =23.49; TE vs WT: p=0.037, F-value =8.48; Late 12-week, df = 94: 6.5m, SC vs WT, p=0.005, F-value =23.34; SC vs TE: p=0.013, F-value =12.94; TE vs WT: p=0.028, F-value =9.81; 8m, SC vs WT, p=0.0043, F-value =24.93; SC vs TE: p=0.009, F-value =16.92; TE vs WT: p=0.031, F-value =11.96; 9.5m, SC vs WT, p=0.0031, F-value =25.99; SC vs TE: p=0.019, F-value =14.29; TE vs WT: p=0.009, F-value =16.17; 24-week, df = 97: 2m, SC vs WT, p=0.011, F-value =19.09; SC vs TE: p=0.015, F-value =17.25; TE vs WT: p=0.33, F-value =2.32; 3.5m, SC vs WT, p=0.008, F-value =21.37; SC vs TE: p=0.009, F-value =19.98; TE vs WT: p=0.41, F-value =2.63; 5m, SC vs WT, p=0.003, F-value =23.09; SC vs TE: p=0.004, F-value =21.17; TE vs WT: p=0.39, F-value =2.95; **Figure 9**.

For the total levels of Nav1.1α, the ANOVA analysis are described as follows: early 12-week, df =97: 2m, SC vs WT, p=0.013, F-value =10.83; SC vs TE: p=0.024, F-value =8.74; TE vs WT: p=0.041, F-value =6.55; 3.5m, SC vs WT, p=0.009, F-value =14.28; SC vs TE: p=0.011, F-value =7.18; TE vs WT: p=0.028, F-value =5.47; 5m, SC vs WT, p=0.000, F-value =20.46; SC vs TE: p=0.001, F-value =17.08; TE vs WT: p=0.014, F-value =11.48; Late 12-week, df = 94: 6.5m, SC vs WT, p=0.006, F-value =14.36; SC vs TE: p=0.019, F-value =8.94; TE vs WT: p=0.033, F-value =4.81; 8m, SC vs WT, p=0.0041, F-value =16.93; SC vs TE: p=0.0082, F-value =13.92; TE vs WT: p=0.027, F-value =7.96; 9.5m, SC vs WT, p=0.0029, F-value =18.99; SC vs TE: p=0.008, F-value =13.29; TE vs WT: p=0.012, F-value =11.17; 24-week, df = 97: 2m, SC vs WT, p=0.016, F-value =18.09; SC vs TE: p=0.025, F-value =11.25; TE vs WT: p=0.036, F-value =4.32; 3.5m, SC vs WT, p=0.013, F-value =20.38; SC vs TE: p=0.02, F-value =13.98; TE vs WT: p=0.031, F-value =7.63; 5m, SC vs WT, p=0.001, F-value =25.01; SC vs TE: p=0.004, F-value =20.17; TE vs WT: p=0.039, F-value =4.68; **Figure 9**.

For the extracellular levels of Nav1.1α, the ANOVA analysis are described as follows: early 12-week, df =97: 2m, SC vs WT, p=0.024, F-value =8.84; SC vs TE: p=0.03, F-value =5.66; TE vs WT: p=0.046, F-value =4.67; 3.5m, SC vs WT, p=0.011, F-value =11.26; SC vs TE: p=0.021, F-value =7.28; TE vs WT: p=0.033, F-value =5.07; 5m, SC vs WT, p=0.026, F-value =9.46; SC vs TE: p=0.031, F-value =7.01; TE vs WT: p=0.04, F-value =4.41; Late 12-week, df = 94: 6.5m, SC vs WT, p=0.007, F-value =14.96; SC vs TE: p=0.015, F-value =10.94; TE vs WT: p=0.022, F-value =9.12; 8m, SC vs WT, p=0.005, F-value =17.91; SC vs TE: p=0.0071, F-value =14.02; TE vs WT: p=0.037, F-value =4.96; 9.5m, SC vs WT, p=0.003, F-value =19.19; SC vs TE: p=0.011, F-value =11.29; TE vs WT: p=0.029, F-value =7.62; 24-week, df = 97: 2m, SC vs WT, p=0.001, F-value =19.09; SC vs TE: p=0.003, F-value =13.25; TE vs WT: p=0.037, F-value =4.88; 3.5m, SC vs WT, p=0.0013, F-value =18.38; SC vs TE: p=0.002, F-value =12.18; TE vs WT: p=0.039, F-value =4.63; 5m, SC vs WT, p=0.001, F-value =22.51; SC vs TE: p=0.0027, F-value =18.18; TE vs WT: p=0.041, F-value =3.13; **Figure 9**.

For the intracellular levels of Nav1.1α, the ANOVA analysis are described as follows: early 12-week, df =97: 2m, SC vs WT, p=0.033, F-value =6.81; SC vs TE: p=0.04, F-value =5.11; TE vs WT: p=0.045, F-value =3.87; 3.5m, SC vs WT, p=0.012, F-value =10.21; SC vs TE: p=0.024, F-value =7.08; TE vs WT: p=0.03, F-value =4.21; 5m, SC vs WT, p=0.019, F-value =9.13; SC vs TE: p=0.021, F-value =7.88; TE vs WT: p=0.034, F-value =4.19; Late 12-week, df = 94: 6.5m, SC vs WT, p=0.004, F-value =16.91; SC vs TE: p=0.009, F-value =12.94; TE vs WT: p=0.031, F-value =7.12; 8m, SC vs WT, p=0.002, F-value =18.22; SC vs TE: p=0.007, F-value =14.18; TE vs WT: p=0.027, F-value =4.06; 9.5m, SC vs WT, p=0.0031, F-value =16.11; SC vs TE: p=0.006, F-value =14.99; TE vs WT: p=0.041, F-value =3.91; 24-week, df = 97: 2m, SC vs WT, p=0.000, F-value =24.91; SC vs TE: p=0.002, F-value =16.25; TE vs WT: p=0.67, F-value =1.18; 3.5m, SC vs WT, p=0.0018, F-value =16.17; SC vs TE: p=0.002, F-value =15.18; TE vs WT: p=0.39, F-value =2.63; 5m, SC vs WT, p=0.0011, F-value =21.03; SC vs TE: p=0.0017, F-value =19.66; TE vs WT: p=0.49, F-value =2.18; **Figure 9**.

There were no significant alterations in Nav1.2 expression in APP/PS1 and WT mice throughout the study, regardless of exercise (data not shown).

**Part 2. For the swimming speed of mice in MWM test, there was no significant difference in speed observed between groups, and the ANOVA analysis are described as follows:** early 12-week, df =97: 2m, SC vs WT, p=0.053, F-value =2.81; SC vs TE: p=0.06, F-value =3.11; TE vs WT: p=0.08, F-value =2.27; 3.5m, SC vs WT, p=0.18, F-value =2.27; SC vs TE: p=0.054, F-value =2.78; TE vs WT: p=0.12, F-value =2.16; 5m, SC vs WT, p=0.72, F-value =1.87; SC vs TE: p=0.061, F-value =2.54; TE vs WT: p=0.21, F-value =1.27; Late 12-week, df = 94: 6.5m, SC vs WT, p=0.28, F-value =1.11; SC vs TE: p=0.07, F-value =3.14; TE vs WT: p=0.32, F-value =1.04; 8m, SC vs WT, p=0.17, F-value =2.97; SC vs TE: p=0.27, F-value =1.24; TE vs WT: p=0.08, F-value =2.32; 9.5m, SC vs WT, p=0.26, F-value =1.57; SC vs TE: p=0.34, F-value =1.21; TE vs WT: p=0.061, F-value =1.09; 24-week, df = 97: 2m, SC vs WT, p=0.07, F-value =3.04; SC vs TE: p=0.25, F-value =1.67; TE vs WT: p=0.87, F-value =1.04; 3.5m, SC vs WT, p=0.09, F-value =2.17; SC vs TE: p=0.13, F-value =2.85; TE vs WT: p=0.27, F-value =1.35; 5m, SC vs WT, p=0.42, F-value =1.31; SC vs TE: p=0.77, F-value =1.24; TE vs WT: p=0.33, F-value =1.18.
